# 

**Published:** 1876-12

**Authors:** 


*CONTENTS.*

| | PAGE | | PAGE |
|---|---|---|---|
| A WORD FOR OURSELVES . . | 493 | DYSPEPSIA . . . . . . . . | 505 |
| OUR NEW STOVE . . . . . | 494 | MAKING A FIRE . . . . . . | 506 |
| THE DIFFERENCE . . . . . | 494 | EXCELSIOR OF AGRICULTURE . | 508 |
| A DICTIONARY . . . . . . . | 495 | EATING AND DRINKING . . . | 509 |
| PERSECUTION . . . . . . . | 496 | WINTER RULES . . . . . . | 511 |
| A BEAUTIFUL PREMIUM . . | 497 | TAKING COLD . . . . . . . | 512 |
| A COOKING SCHOOL . . . . | 497 | CONSTIPATION . . . . . . . | 513 |
| GAIL BORDEN'S GREAT INVENTIONS . . . . . . . . . | 498 | CIVILIZATION AND LONGEVITY | 514 |
| DR. JACKSON'S GRANULA . . | 498 | BUCKWHEAT CAKES . . . . | 515 |
| WARWICK MINERAL SPRING . | 498 | PORK AS FOOD . . . . . . | 516 |
| BOOK NOTICES . . . . . . . | 500 | WEARING FLANNEL . . . . | 518 |
| AWAY WITH COSMETICS . . . | 500 | THE HUMAN HAIR . . . . . | 519 |
| THE HEALTH FOOD COMPANY . | 501 | GROWLERS . . . . . . . . . | 525 |
| PERTAINING TO INSURANCE . | 502 | SELF-MEDICATION . . . . . | 526 |
| | | WARMING HOUSES . . . . . | 527 |

